# Inclusion of women in clinical trials

**DOI:** 10.1186/1741-7015-7-56

**Published:** 2009-10-09

**Authors:** Jesse A Berlin, Susan S Ellenberg

**Affiliations:** 1 Johnson & Johnson Pharmaceutical Research and Development, Titusville, NJ, USA; 2 University of Pennsylvania School of Medicine, Philadelphia, PA, USA

## Abstract

There is increasing concern among many in the medical arena about the extent to which the effects of treatment, either good or bad, apply to specific subgroups of individuals. Women comprise one of the most frequently considered 'subgroups' of patients. In the 1980s, much political attention was focused on concerns about equity in the research enterprise. In this paper, we briefly describe the statutory approaches to achieving equity in research, beginning with The NIH Revitalization Act of 1993. We go on to describe clinical, methodological and political factors affecting these discussions. We conclude that the controversy over the inclusion of women in clinical trials probably stems, in part, from theoretical concerns about gender differences in treatment effects and, in part, by legitimate fears of exposing fetuses to investigational drugs. However, we believe that the broader issue centres on biological factors, possibly defined by genes or gene expression, that may directly or indirectly modify the effect of specific treatments on specific individuals.

A growing concern of physicians, regulators, healthcare policy makers and patients is the extent to which the effects of treatment, both good and bad, apply to specific subgroups. Do results of clinical trials apply consistently and equally across all clinically meaningful subclasses of patients enrolled in the studies? Can the results of those studies be extrapolated to patients or types of patients who did not participate in the original research? Reliable data on these issues are rarely available at the time of drug approval and are more difficult to generate once the drug is on the market and readily available.

## Inclusion of women in trials

Women comprise one of the most frequently considered 'subgroups' of patients for many medical conditions. Beginning in the 1980s, a great deal of political attention was focused on the concerns about equity in the research enterprise. Trials, such as the Multiple Risk Factor Intervention Trial [1,2] and the Physicians' Health Study [3,4], both primary prevention trials, studied only men. This was because of the lower incidence of cardiovascular disease among middle-aged women, the desire to minimize heterogeneity of the trial population and, in the case of the Physician's Health Study, the small proportion of female physicians available at the time that the study was undertaken. These practical considerations relied on the underlying assumption that the treatment effects in women would be similar to those in men, an assumption that became increasingly challenged [5-7].

Meinert and colleagues [8] argued that there was little evidence to support the perception that women were underrepresented in trials. Even in trials for heart disease, nearly 68% involved both men and women These authors suggested that in heart disease, as well as in HIV, the directions of the male-female differences are consistent with the burden of disease. They further noted that for neoplasms, female-only trials (20.8%) outnumbered male-only trials (8.7%) by a substantial margin.

Nevertheless, concerns about the exclusion of women from some trials of important medical issues led to the NIH Revitalization Act of 1993 [9] The Act mandated that NIH-funded trials have sample sizes adequate to support a 'valid analysis' of gender and racial subgroup effects. The statute allows exceptions to the requirement for entering women and minorities when there is substantial evidence of the lack of difference in effects of treatment between subgroups.

While the 1993 legislation was primarily motivated by concerns about understanding the effects of treatment on women, controversies were brewing for other reasons. Women with child-bearing potential had long been excluded from clinical trials due to concerns about exposing fetuses to experimental drugs. This convention was challenged in the 1990s when HIV-infected women found themselves ineligible to participate in trials of new antiviral therapies; at a time when very few such therapies were available, the potential benefits of having access to a new treatment for this life-threatening disease appeared to most women to outweigh any possible risks [7]. In response to these concerns, the FDA took formal steps to facilitate the inclusion of premenopausal women into clinical trials under certain circumstances [10].

A related, and even more challenging problem, was the treatment of pregnant women. There is a widespread reluctance to expose pregnant women to investigational drugs because of possible risks to developing fetuses. The implication of this situation, however, is that pregnant women who get sick, who have chronic diseases requiring maintenance medication or who face a risk (for example, who possibly need a new vaccine) are unable to weigh the potential benefits against the potential risks, since there will be no data on treatment effects in pregnant women. Pregnant women who must take certain medications are essentially participating in an uncontrolled and unmonitored experiment for which the data will most likely never be assessed. A possible exception may be trials of the H1N1 flu vaccine; pregnant women are considered part of the high-risk group for this infection and there have been indications that pregnant women will be included in later cohorts of the clinical trials [11].

## Methodological Issues in Assessing Treatment Effects in Subgroups

The exploration of treatment effects in patient subgroups has been controversial. Most trials are not designed with sample sizes that are large enough to detect moderate interactions in treatment effects among subgroups or to develop precise estimates of the effects within subgroups. Meta-analysis of completed clinical trials may be useful for exploring these questions with improved statistical power, but often there are no multiple studies that are sufficiently similar to support an informative meta-analysis.

Freedman and colleagues [12], in their commentary on the 1993 legislation described above, stress the importance of using appropriate methods to compare intervention effects among gender and racial/ethnic subgroups. They stress the possibilities of finding clinically unimportant but statistically significant differences, and *vice versa*. In fact, they go on to argue against designing trials with sufficient power to detect treatment by subgroup interactions in the absence of *a priori *evidence that such subgroup differences might exist. (How one generates this *a priori *evidence is left unclear.) Clinical trialists recognize that the requirement that all studies should be powered statistically to detect treatment X gender (or other) interactions would make trials infeasible. Freedman *et al*. also suggest that meta-analysis of multiple trials is the best way to obtain reliable information about subgroup differences.

Differences in treatment effect across subgroups of patients do not all have the same implications. The type of difference that might cause the most concern would be a directional difference: one group benefits from the treatment while the other is harmed. Such differences are rare; when they have been occasionally suggested they have generally not been supported by other data [13-15]. It would also be important to be aware of a difference in magnitude of effect if the difference was substantial, as it would affect risk-benefit considerations. There are a few examples of this phenomenon. One recent example, of a heart failure drug that appeared effective in African Americans but ineffective in whites, remains controversial but is supported by data from multiple studies [16,17].

The NIH Office of Research in Women's Health maintains data on the inclusion of women in NIH-funded trials. The most recent report shows that women comprise approximately 55% of clinical trial participants [18]. Overall numbers, however, may be less informative than they seem. For example, although the annual incidence of breast cancer is about the same as that for prostate cancer, enrollment in breast cancer trials is much higher than in prostate cancer trials [19]. Many other diseases are not gender neutral; more women get autoimmune diseases; more boys are diagnosed with ADHD. When there are exciting new molecules to study in a given medical area, trials in this area may have a greater advantage, thereby contributing to an artifactual gender imbalance in trial participants. These confounding issues, which may have their own time trends, make it difficult to assess the extent to which men and women are proportionally represented in trials of disease affecting both genders.

In summary, controversy over the inclusion of women in clinical trials has been motivated, in part, by theoretical concerns about gender differences in the effect of the treatment and, in part, by legitimate fears of exposing fetuses to investigational drugs. There is no question that some treatments do work differently in men and women, but the proportion of treatments for which men and women respond very differently is unknown. The broader issue really centres on biological factors, possibly defined by genes or gene expression, that may directly or indirectly modify the effect of specific treatments on specific individuals. Whether the current explosive interest in genetic profiling will ultimately lead to the medical nirvana of personalized medicine, that many have predicted, remains to be seen.

## Competing interests

JAB is a full-time employee of Johnson & Johnson Pharmaceutical Research and Development, which financed the article-processing costs. He is unaware of any direct or indirect gain or loss that might result from publication of this commentary. SSE has no equity in any for-profit company. She receives compensation from several pharmaceutical companies for consultation and service on data monitoring committees but does not believe these represent competing interests with regard to the content of this manuscript.

## Authors' contributions

JAB drafted the initial version of the manuscript. SSE made extensive revisions and additions to that initial draft. Both authors read and approved the final manuscript.

## Pre-publication history

The pre-publication history for this paper can be accessed here:


